# Acute Dysnatremias - a dangerous and overlooked clinical problem

**DOI:** 10.1186/s13049-019-0633-3

**Published:** 2019-05-28

**Authors:** David Joergensen, Kiarash Tazmini, Dag Jacobsen

**Affiliations:** 1 Department of Acute Medicine, Division of Medicine, Oslo University Hospital, NO-0454 Oslo, Norway; 2 Department of Internal Medicine, Diakonhjemmet Hospital, PO BOX 23 Vinderen, 0319 Oslo, Norway; 3 Department of Endocrinology, Morbid Obesity and Preventive Medicine, Faculty of Medicine, Oslo University Hospital, Postbox 4950 Nydalen, 0424 Oslo, Norway; 4 Department of Acute Medicine, Oslo University Hosptial and University of Oslo, Oslo, Norway; 5 National Poisons Information Centre, Oslo, Norway

**Keywords:** Acute dysnatremia, Hyponatremia, Hypernatremia, Water intoxication, Salt intoxication, Psychogenic polydipsia, Exercise-associated hyponatremia, Ecstasy-associated hyponatremia

## Abstract

**Background:**

Dysnatremias are common electrolyte disturbances with significant morbidity and mortality. In chronic dysnatremias a slow correction rate (<10 mmol/L/24 h) is indicated to avoid neurological complications. In acute dysnatremias (occurring <48 h) a rapid correction rate may be indicated. Most guidelines do not differ between acute and chronic dysnatremias. In this review, we focus on the evidence-based treatment of acute dysnatremias.

**Methods:**

A literary search in PubMed and Embase. A total of 72 articles containing 79 cases were included, of which 12 cases were excluded due to lack of information.

**Results:**

Of 67 patients (70% women) with acute dysnatremia, 60 had hypo- and 7 had hypernatremia. All patients with hyper- and 88% with hyponatremia had a rapid correction rate (> 10 mmol/L/24 h). The median time of correction was 1 day in patients with hypo- and 2.5 days in patients with hypernatremia. The mortality was 7% in patients with hypo- and 29% in patients with hypernatremia.

**Interpretation:**

Severe acute dysnatremias have significant mortality and require immediate treatment. A rapid correction rate may be lifesaving and is not associated with neurological complications. Chronic dysnatremias, on the other hand, are often compensated and thus less severe. In these cases a rapid correction rate may lead to severe cerebral complications.

**Electronic supplementary material:**

The online version of this article (10.1186/s13049-019-0633-3) contains supplementary material, which is available to authorized users.

## Background

Dysnatremias are common electrolyte disturbances with significant morbidity and mortality [1, 2].

Hyponatremia (<135 mmol/L) has a prevalence of 15–30% [1, 3, 4], and hypernatremia (> 145 mmol/L) a prevalence of 9% [3]. Most cases are chronic, and common causes of chronic hyponatremia include medications; especially thiazide-diuretics, heart failure, advanced liver- and renal disease and syndrome of inappropriate antidiuretic hormone secretion (SIADH) [1, 4]. Chronic hypernatremia is often caused by loss of free water or a diet rich in salt [2].

Acute dysnatremias (<48 h) are usually caused by excessive intake of water or salt within a short period of time. Renal and extra-renal water loss, as well as salt loss or retention usually takes more time to develop. Groups at risk include psychiatric patients, children, elderly with reduced cognitive functions and endurance athletes.

Severe dysnatremias, usually defined as serum-sodium < 125 mmol/L or > 165 mmol/L, are associated with neurological signs and symptoms with increased mortality. Nausea, falling and asthenia are common in severe hyponatremia while decreased consciousness and confusion are common in severe hypernatremia. Other signs and symptoms include headache, dizziness, agitation and seizures [5]. In severe hyponatremia, the prognosis depends on the underlying condition and a number of other factors, including the rate of correction [3]. Hypernatremia has a mortality of 40–60% [2]. Since most dysnatremias are chronic, there is insufficient data to estimate prevalence and mortality in acute dysnatremias, but the mortality in salt intoxications with serum-sodium > 170 mmol/L is close to 100% [6]. The significance of rapid correction rates on the survival of acute dysnatremias are also unknown.

In chronic dysnatremias a slow correction rate (< 10 mmol/L/24 h) is preferred to avoid neurological complications [1]. There is usually a compensated osmolality disorder across the blood-brain barrier and rapid changes in osmolality may be dangerous. Current guidelines [1, 4, 7] mainly focus on the treatment of chronic dysnatremias. With this review, we question the practice of treating acute dysnatremias with a slow correction rate, as applied pathophysiology and a few published cases suggests that a rapid correction may be lifesaving.

## Methods

This review is based on a search in PubMed and EMBASE. The search had no time limit and was ended April 6th 2017.

Search terms were acute OR severe AND hyper-, hypo-, dysnatremia, polydipsia, water intoxication, salt poisoning OR exercise-associated hyponatremia, both as MeSH (Medical Subject Heading) in PubMed and EMTREE Subject Heading in EMBASE and as regular words in title and abstracts in both databases. We used filters for language and age groups. Complete search string is available in the Additional file 1.

The search resulted in 5413 articles (1743 in PubMed and 3670 in EMBASE). After the removal of doublets 3884 articles remained. Relevant articles were selected based on title and abstract. Inclusion criteria: Adults ≥18 years old and duration of symptoms <48 h before admission. Patients developing hypo- or hypernatremia due to treatment in hospital were excluded. As were articles lacking information about the treatment given or the correction rate, some of these are still cited in the discussion. All articles were in English, Norwegian, Swedish or Danish. We included 263 articles and 72 articles were read in full text.

## Results

**Fig. 1:**
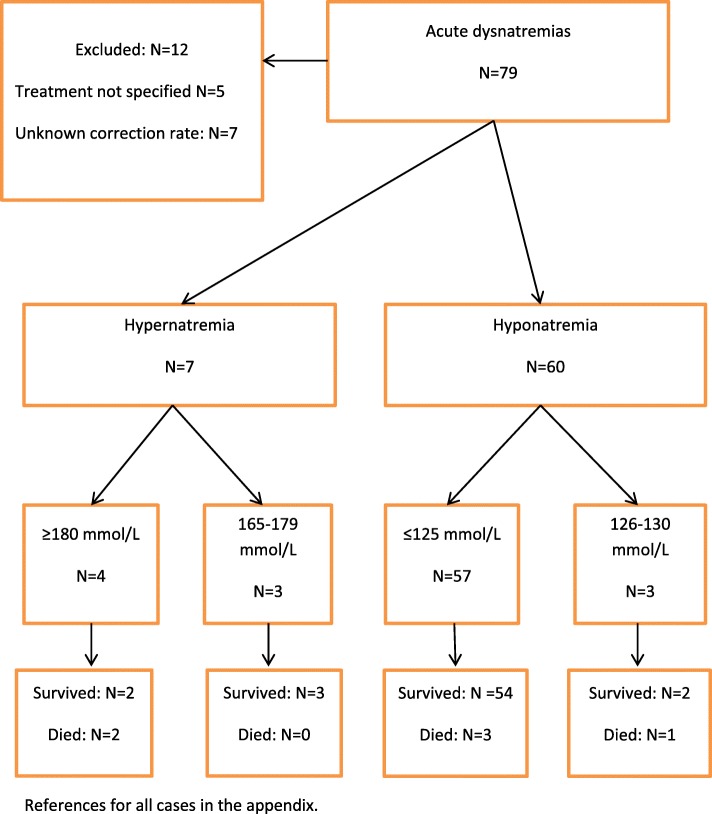
Study flow-chart in 79 patients with acute dysnatremias

The search resulted in 72 articles, containing 79 cases of whom 12 were excluded due to lacking information about treatment and correction rate. The flow chart (Fig. 1) and Table 1 show 67 cases of acute dysnatremia, of which 60 had hypo- and 7 had hypernatremia. Median serum-sodium was 116 mmol/L in hyponatremia and 196 mmol/L in hypernatremia. Lowest serum-sodium was 98 mmol/L and highest was 246 mmol/L.

**Table 1:** Acute dysnatremias^a^

	Hyponatremia						Hypernatremia			Dysnatremia
	Total	Other over hydration	Exercise-associated	Primary polydipsia	Other	Ecstasy-associated	Total	Salt intoxication	Other	Total
Number (%)	60 (90)	23 (34)	14 (21)	9 (13)	8 (12)	6 (9)	7 (11)	4 (6)	3 (5)	67 (100)
Sex (women) Number (%)	43 (72)	20 (87)	6 (43)	4 (44)	7 (88)	6 (100)	4 (57)	2 (50)	2 (67)	47 (70)
Age (years) Median (lower-upper quartile)	41 (29–54)	41 (32–59)	45 (32–53)	32 (25–36)	51 (46–69)	19,5 (19–22)	36 (20–52)	28 (20–39)	52 (35–66)	41 (27–53)
S-sodium (mmol/l) at hospital arrival, median (lower-upper quartile)	116 (111–122)	120 (115–122)	120 (115–122)	108 (104–112)	108 (101–112)	116 (115–120)	196 (174–209)	203 (185–228)	175 (169–206)	118 (111–122)
GCS < 15, number (%)	46 (77)	15 (65)	11 (79)	7 (78)	7 (88)	6 (100)	5 (71)	3 (75)	2 (67)	51 (76)
Seizures, number (%)	40 (67)	14 (61)	11 (79)	6 (67)	6 (75)	3 (50)	1 (14)	1 (25)	–	41 (61)
Correction rate, number (%)										
Slow ≤ 10 mmol/l/24 h	7 (12)	4 (17)	2 (14)	0	1 (13)	0	0	0	0	7 (10)
Rapid > 10 mmol/l/24 h	53 (88)	19 (83)	12 (86)	9 (100)	7 (88)	6 (100)	7 (100)	4 (100)	3 (100)	60 (90)
Mortality, number (%)	4 (7)	3 (13)	1 (7)	0	0	0	2 (29)	2 (50)	0	6 (9)

^a^Due to rounding of percentage, the total is not 100%

Almost all, 91% (61/67), had onset of signs and symptoms <24 h before admission. In hyponatremia, 77% (46/60) had decreased consciousness and 67% (40/60) had seizures (Table 1). In hypernatremia, 71% (5/7) had decreased consciousness and 14% (1/7) had seizures. Other signs and symptoms of increased intracranial pressure such as headache, nausea and vomiting were reported in 49% of cases. Other neurological signs and symptoms were reported in 55% of cases.

In patients with hyponatremia, 50% (30/60) were treated with hypertonic saline infusions, 33% were given isotonic infusions, one patient was given hypertonic infusions and dialysis, and one was not given saline but mannitol 20% infusion. The rest were treated with fluid restriction. In patients with hypernatremia, 86% (6/7) received hypotonic infusions. One were given isotonic infusions and water by nasogastric tube.

Median time of correction was 1 [1, 2] day in hyponatremia and 2.5 [1–3] days in hypernatremia.

Table 2 shows the 6 fatal cases of which 4 had hypo- and 2 hypernatremia. Lowest serum-sodium at hospital arrival was 106 mmol/L and highest 246 mmol/L. A rapid correction rate (> 10 mmol/L/24 h) was attempted, but all patients died within hours or a few days after hospital arrival.

**Table 2:** Acute dysnatremias and mortality sorted by increasing serum-sodium

Patient	Sex	Age	Serum-sodium (mmol/l) at hospital arrival	Duration of symptoms at arrival	Cause	Neurological signs/symptoms	Treatment given	Correction of s-sodium first 24 h	Time to death
1	Female	27	106	30 min.	Over hydration and gastroenteritis last 14 h.	Coma, seizures.	Hypertonic NaCl and furosemide.	21 mmol/l during 7 h.	Declared brain dead 16 h after arrival.
2	Male	18	121, falling to 115 before transfer to ICU.	6 h.	Water intoxication estimated to 20 L in 6 h.	Nausea, headache, confusion, decreased consciousness.	NaCl 9 mg/ml and mannitol.	30 mmol/l during 12 h.	Derived diabetes insipidus, sepsis and DIC, died within few days.
3	Female	20	123	1–2 h.	Over hydration before urinary sample, estimated to 10 L within 2–3 h.	Headache, dizziness, seizures, decreased consciousness.	NaCl 9 mg/ml and furosemide.	21 mmol/l during 18 h.	2 days.
4	Female	47	127	2 h and 45 min.	Exercise-associated hyponatremia.	Headache, vomiting, decreased consciousness (GCS 7).	NaCl 9 mg/ml and 3%, also mannitol and furosemide.	16 mmol/l during 4 h.	19 h after onset of symptoms.
5	Male	41	209	4 h.	Salt intoxication, drank mouthwash.	Status epilepticus, coma (GCS 3).	Glucose 50 mg/ml, NaCl 4,5 mg/ml and NaCl 9 mg/ml	18 mmol/l during 13 h, 27 mmol/l during 29 h.	72 h after intoxication.
6	Female	36	246	< 24 h.	Salt intoxication during excorcism.	Coma.	NaCl 9 mg/ml, glucose 50 mg/ml and furosemide.	53 mmol/l during 7 h.	Clinically brain dead 5 h after arrival.

## Pathophysiology

To maintain homeostasis, cell function and size, the physiological regulation of osmolality is important. Osmolality is regulated by the intake and excretion of water and salts. Cell membranes contain water channels (aquaporines) and water diffuses freely between the extracellular and intracellular space, implicating that extra- and intracellular osmolality is approximately the same. Changes in serum osmolality (normal range 275–300 mosmol/kg/H_2_O) will thus, uncompensated, lead to a change in cell size. In physiological osmoregulation, thirst and the secretion of antidiuretic hormone (ADH) is suppressed by serum-sodium approximately < 135 mmol/L, and is stimulated by serum-sodium approximately > 135 mmol/L [8]. In addition to high osmolality, ADH-secretion is stimulated by hypovolemia, sympathetic activity (pain, nausea, stress, anxiety) and some drugs [1, 9].

Sodium is quantitatively the most important factor determining serum osmolality. Hypernatremia leads to increased serum osmolality (hypertonicity), while hyponatremia in most cases leads to decreased serum osmolality (hypotonicity) [8]. Exceptions occur in the presence of “effective” osmoles as glucose, mannitol and contrast agents. Urea and alcohols are “ineffective” osmoles, increasing serum osmolality without causing hyponatremia. Pseudohyponatremia is a laboratory artefact no longer occurring with the use of ion-selective electrodes [1].

Serum-sodium will fall rapidly if the amount of water intake, or hypotonic fluids given intravenously, exceeds the renal capacity of water excretion. In healthy adults a water intake of 1 L/hour may lead to hyponatremia [10]. In case of reduced renal function, a lower water intake will be sufficient to cause hyponatremia [11].

Conversely, serum-sodium will rise rapidly if concentrated salt (sodium-chloride) is eaten or given intravenously. Loss of electrolyte-free water due to aquaresis or osmotic diuresis, that is not replaced, will also cause increased serum-sodium [8].

Due to its specialized function and the space limiting anatomy of the skull, the brain is particularly vulnerable to changes in osmolality and cell size. Brain cells continuously compensate gradual changes in extracellular osmolality by pumping in, or out, electrolytes and endogenous osmolytes [9]. Sustained changes in osmolality involves a change in transport and metabolism of these. This may to some extent compensate for the change in osmolality across the blood-brain barrier in chronic dysnatremias [8, 9]. This compensation develops in (24-) 48 h [1, 8] and thus 48 h is used as a distinction between acute and chronic dysnatremias. A too rapid correction of chronic hyponatremia will induce a hypertonic stress, while a too rapid correction of chronic hypernatremia will induce a hypotonic stress. In chronic (and partly or fully compensated) dysnatremias a too rapid correction may be more dangerous than the actual disorder itself.

## Main causes of acute dysnatremias

**Primary polydipsia** is a condition with increased thirst and water intake, often secondary to psychiatric disorders occurring in 20–47% of schizophrenic patients of whom up to 7% develop hyponatremia [4, 11]. Many psychotropic medications are associated with SIADH [1] increasing the risk of this patient group.

**Water intoxication** without psychiatric cause is reported in advance of urinary samples [12], medical examinations [13], concurring infections [14], and contests like water poker and others in which intake of 5–6 L of water in a short period of time has been fatal [15].

**Ecstasy-associated hyponatremia** is complicated and due to large water intake, often secondary to ecstasy mediated hyperthermia with loss of water and electrolytes, and non-osmotic ADH-secretion mediated by ecstasy and its metabolites [11, 16]. Women are overrepresented (85%) [11].

**Exercise-associated hyponatremia** (EAH) is defined as hyopnatremia occuring during, or within 24 h of prolonged physical activity [17]. Severe cases occur in 2–7% of participants in endurance sports [11, 18]. Two major pathologic mechanisms largely account for the development of EAH; excessive fluid intake and non-osmotic secretion of ADH secondary to physical activity, pain, nausea, vomiting or hypoglycemia [17, 19]. Specific guidelines for EAH recommends oral administration of salt to symptomatic patients. In severe cases intravenous bolus of hypertonic saline (3%) is recommended, if available [17, 19].

The treatment of severe cases of primary polydipsia, water intoxication and ecstasy-associated hyponatremia will be the same, namely intravenous bolus of hypertonic (3%) saline, or salty (NaCl) items given orally. The treatment goal is not full correction, but counteracting cerebral edema. A rapid increase in serum sodium of 4–6 mmol/L has been shown to be sufficient in most patients [4]. With normal diuresis serum-sodium will then normalize quickly, although some may need more saline. If chronic hyponatremia is suspected, or in patients with unknown duration, the immediate treatment is the same but the daily correction rate is limited to < 10 mmol/L/24 h [1, 4].

**Salt intoxication** occurs in attempts of suicide, to induce emesis, or when salt is mistaken for sugar. Groups at risk include psychotic patients, children, disabled people and elderly with dementia. Campbell et al. [20] found 35 salt intoxications with fatal outcome. Lethal dose of salt (NaCl) was estimated to 25 g (approximately 400 mmol; < 5 tea spoons) in children and 60 g (approximately 1000 mmol; < 4 table spoons) in adults. The treatment is large amount of water orally, or rapid intravenous infusions of glucose 50 mg/ml, to compensate for extracellular hypertonicity. The latter may cause hyperglycemia and serum-sodium should be corrected for hyperglycemia. Further treatment consists of loop diuretics in order to reduce the created isosmotic fluid overload.

## Discussion

Most acute dysnatremias are hyponatremias with onset of symptoms <24 h before hospital arrival. Decreased consciousness and seizures are common, and women are overrepresented. In most patients serum-sodium is corrected faster than recommended for chronic dysnatremias. Acute hypernatremias have a higher mortality than acute hyponatremias.

The overrepresentation of women among patients with acute hyponatremias, including exercise-associated hyponatremia [19] and ecstacy-associated hyponatremia [11], may be caused by the fact that women in general have less body water, both because of lower body weight and higher fat percentage. The addition of the same amount of free water, or salt, will thus induce a larger change in serum-sodium. Estrogen may also contribute [21].

In patients who died of acute hypernatremia a rapid correction was attempted, but the patients died within a short period of time. Among patients excluded due to lacking information about treatment given or correction rate, the mortality was 3/12 (25%). All fatal salt intoxications (serum-sodium 175–255 mmol/L) happened within 4–36 h after hospital arrival. We also excluded several other cases due to lacking information about duration of symptoms, including several autopsy reports. Our numbers is thus not representative for the actual mortality in acute dysnatremias.

Most patients with hyponatremia, and all with hypernatremia, had faster correction rates than 10 mmol/L/24 h. A significant proportion were corrected as fast as 20–50 mmol/L/24 h. This is up to five times as fast as most guidelines recommend in chronic dysnatremias [1, 22, 23]. However some experts recommend a faster correction rate in acute and symptomatic patients; Sterns recommends up to 1 mmol/hour, with no upper 24-h limit in acute hyponatremias, and that acute hypernatremia with known duration under 24 h is corrected as fast as possible [8]. Verbalis has no upper limit for the correction rate of acute hyponatremia [4].

Among the surviving patients with the fastest correction rate, the following are emphasized: a young man with serum-sodium 196 mmol/L received 6 l of glucose solution intravenously, within half an hour, with a documented drop in serum-sodium of 37 mmol/L [24]. A man with acute hyponatremia, 98 mmol/L, received dialysis and hypertonic saline with an increase in serum-sodium of 24 mmol/L in 5 h [25]. Considering the short survival time in the fatal cases, these rapid correction rates may have been lifesaving.

**Fig. 2:**
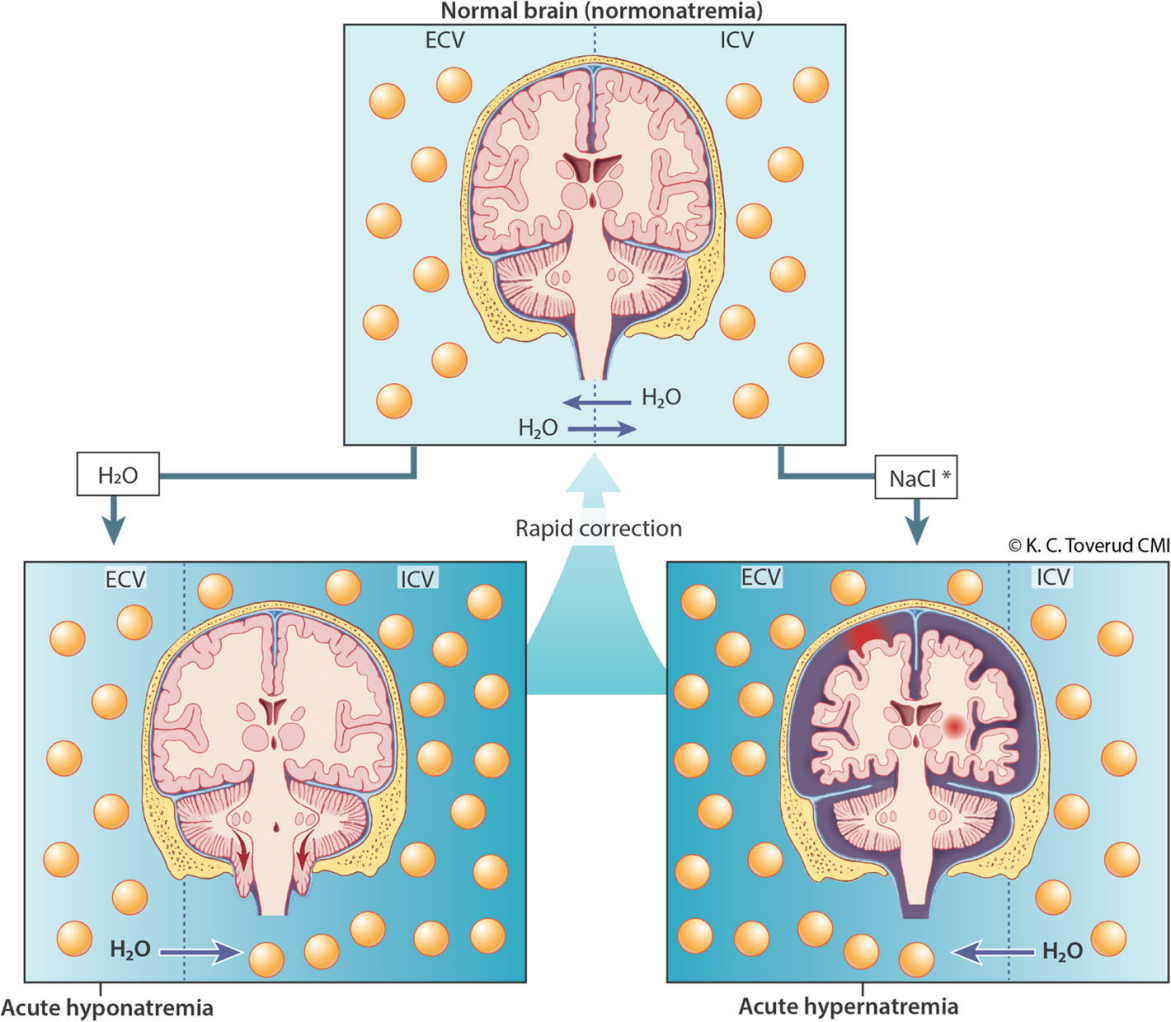
Dysnatremia and the effects on the brain. In normal condition, there is osmotic equilibrium. Water diffuses between the extracellular volume (ECV) and intracellular volume (ICV). In acute hyponatremia, water from ECV diffuses into ICV (water is drawn into cells), which can lead to brain edema and herniation. In acute hypernatremia, water from ICV diffuses into ECV (water is extracted from the cells), which can lead to reduced brain volume. This can cause rupture of cerebral veins, focal and subarachnoid bleeding. Upon rapid correction of acute hyponatremia or acute hypernatremia, the brain can be returned to normal condition (normonatremia)

A difficult clinical challenge is to distinguish between acute and acute-on-chronic hyponatremia in patients with hyponatremic encephalopathy. It also occurs that assumed acute hyponatremia has underlying causes (such as heart-, liver-, or renal disease or the use of certain drugs) and thus also may have a chronic (and partly compensated) component. These patients may get complications if a rapid correction rate is used. Our experience is that a head CT with signs of cerebral edema supports an acute genesis (Fig. 2). Both European and American guidelines for hyponatremia [1, 4] recommend that all symptomatic patients receive intravenous bolus doses of hypertonic (3%) saline. However, we question the recommendations given in the European guidelines [1] regarding the maximum correction rate of 10 mmol/L/24 h in the treatment of acute hyponatremia with known duration <48 h and no underlying risk factors.

### Limitations

Although the search resulted in 3884 articles, only 67 cases made our inclusion criteria. Many articles and cases were excluded due to unknown duration of symptoms or underlying risk factors and/or insufficient information about the treatment given, or the correction rate. For the same reason no reviews or randomized controlled studies were included. Although some larger studies have compared survival and correction rate in dysnatremias, these do not distinguish clearly between acute and chronic dysnatremias. To our knowledge no randomized controlled trial regarding the correction rate of acute dysnatremias has been published.

In this review, we did not have access to the hour-by-hour correction rate for all patients. Thus we cannot know if the patients had a rapid correction of serum-sodium which then decreased, or if they had a steady correction rate. Publication bias may have contributed to the high survival and the rapid correction rate in our material. However, we believe it is worth noticing the survival- and correction rate in context with the fact that none of the authors reported signs or symptoms consistent with osmotic demyelination syndrome. Only a small number of patients received a follow-up MRI, thus we cannot rule out that some patients may have had radiological deviations, but in our review we have not found any cases of osmotic demyelination syndrome secondary to rapid correction of acute hyponatremia.

## Conclusions

Severe acute dysnatremias have high mortality and require immediate treatment. Many guidelines, textbooks and reviews recommend a correction rate of 0.5–1.0 mmol/L/h, with a maximum correction of 8–12 mmol/L/24 h [1, 22, 23]. This implies that serum-sodium is corrected in 2–3 days – if the patient survives that long. Applied pathophysiology and expert recommendations by Verbalis [4] and Sterns [8] suggests that patients with acute dysnatremias have lower risk of complications due to rapid correction than patients with chronic hyponatremia. Our review supports this, and it may be that a more rapid correction rate may be lifesaving in selected patients. However, our review is based on a limited number of cases and further research is needed to distinguish between treatments of acute and chronic dysnatremias.

## Additional file

Additional file 1: Acute Dysnatremias. (DOCX 247 kb)

## Abbreviations

ADH: Antidiuretic hormone
CT: Computed tomography
EAH: Exercise-associated hyponatremia
ECV: Extracellular volume
GCS: Glasgow coma scale.
ICV: Intracellular volume
MRI: Magnetic resonance imaging
SIADH: Syndrome of inappropriate antidiuretic hormone secretion
