# Supplementary material for: Acute Dysnatremias - a dangerous and overlooked clinical problem
Source: Scand J Trauma Resusc Emerg Med. 2019 May 28;27:58. doi: 10.1186/s13049-019-0633-3 (PMC6540386; doi:10.1186/s13049-019-0633-3)
Supplement: Supplementary file 1 — Acute Dysnatremias. (DOCX 247 kb) [file 13049_2019_633_MOESM1_ESM.docx]

Additional file 1-Acute Dysnatremias

**Complete search string:**

We used the search: acute OR severe AND hyper-, hypo-, dysnatremia, polydipsia, water intoxication, salt poisoning OR exercise-associated hyponatremia both as MeSH (Medical Subject Heading in PubMed) and as EMTREE (in EMBASE), and as regular words in title and abstracts in both databases.

The search was ended April 6. 2017. Both ways of spelling natremia were used (ie. natremia or natraemia). The results were limited with filters for language (English, Danish, Norwegian and Swedish), and age «Adult: 19+ years, Adolescent: 13-18 years» in PubMed, and «adult <18 to 64 years> or aged <65+ years>» in EMBASE.

Search: (((((((((((hyponatremia[MeSH Terms]) AND exercise-associated[Title/Abstract])) OR ((((salt poisoning[Title/Abstract]) OR water intoxication[Title/Abstract])) OR Water Intoxication[MeSH Terms])) OR (((((dysnatremia[Title/Abstract]) OR (((hypernatremia[MeSH Terms]) OR hypernatremia[Title/Abstract]) OR excessive sodium[Title/Abstract])) OR ((sodium depletion[Title/Abstract]) OR ((hyponatremia[MeSH Terms]) OR hyponatremia[Title/Abstract])))))) OR Polydipsia, Psychogenic[MeSH Terms]) AND (((((acute[Title/Abstract]) OR severe[Title/Abstract])))) Filters: Humans, English, Norwegian, Danish, Swedish, Adult: 19+ years, Adolescent: 13-18 years

**Flow chart with references:**

126-130 mmol/l

N=3

≤125 mmol/l

N=57

165-179 mmol/l

N=3

≥180 mmol/l

N=4

Hyponatremia

N=60

Hypernatremia

N=7

Excluded: N=12

Treatment not specified: N=5(8-12)

Unknown correction rate: N=7(11, 65-69)

Acute dysnatremias

N=79

Survived: N=2 (1, 2)

Died: N=2 (13, 14)

Survived: N=54 (3, 12, 16-65)

Died: N=3 (70-72)

Survived: N=2 (3, 4)

Died: N=1 (15)

Survived: N=3 (5-7)

Died: N=0

1. Carlberg DJ, Borek HA, Syverud SA, Holstege CP. Survival of acute hypernatremia due to massive soy sauce ingestion. J Emerg Med. 2013;45(2):228-31.

2. Park YJ, Kim YC, Kim MO, Ruy JH, Han SW, Kim HJ. Successful treatment in the patient with serum sodium level greater than 200 mEq/L. J Korean Med Sci. 2000;15(6):701-3.

3. Ellis C, Cuthill J, Hew-Butler T, George SM, Rosner MH. Case report: exercise-associated hyponatremia with rhabdomyolysis during endurance exercise. Phys Sportsmed. 2009;37(1):126-32.

4. Kott E, Marcus Y. Acute brain edema due to water loading in a young woman. Eur Neurol. 1985;24(4):221-4.

5. Ellis RJ. Severe hypernatremia from sea water ingestion during near-drowning in a hurricane. West J Med. 1997;167(6):430-3.

6. Ju HJ, Bae HJ, Choi DE, Na KR, Lee KW, Shin YT. Severe hypernatremia by excessive bamboo salt ingestion in healthy young woman. Electrolyte and Blood Pressure. 2013;11(2):53-5.

7. Ward MJ, Routledge PA. Hypernatraemia and hyperchloraemic acidosis after bleach ingestion. Hum Toxicol. 1988;7(1):37-8.

8. Kwon C, Zaritsky A, Dharnidharka VR. Transient proximal tubular renal injury following Ecstacy ingestion. Pediatr Nephrol. 2003;18(8):820-2.

9. Machino T, Yoshizawa T. Brain shrinkage due to acute hypernatremia. Neurology. 2006;67(5):880.

10. Mor F, Mor-Snir I, Wysenbeek AJ. Rhabdomyolysis in self-induced water intoxication. J Nerv Ment Dis. 1987;175(12):742-3.

11. Turk EE, Schulz F, Koops E, Gehl A, Tsokos M. Fatal hypernatremia after using salt as an emetic--report of three autopsy cases. Leg Med (Tokyo). 2005;7(1):47-50.

12. Yalcin-Cakmakli G, Oguz KK, Shorbagi A, Bas DF, Ergan-Arsava B, Kunt M, et al. Hyponatremic encephalopathy after excessive water ingestion prior to pelvic ultrasound: Neuroimaging findings. Intern Med. 2010;49(16):1807-11.

13. Raya A, Giner P, Aranegui P, Guerrero F, Vazquez G. Fatal acute hypernatremia caused by massive intake of salt. Arch Intern Med. 1992;152(3):640, 6.

14. Moder KG, Hurley DL. Fatal hypernatremia from exogenous salt intake: Report of a case and review of the literature. Mayo Clin Proc. 1990;65(12):1587-94.

15. Myers TM, Hoffman MD. Hiker Fatality From Severe Hyponatremia in Grand Canyon National Park. Wilderness Environ Med. 2015;26(3):371-4.

16. Ajaelo I, Koenig K, Snoey E. Severe hyponatremia and inappropriate antidiuretic hormone secretion following ecstasy use. Acad Emerg Med. 1998;5(8):839-40.

17. Baskar V, Kamalakannan D, Singh BM. Carbamazepine-induced rapid and severe hyponatraemia. Pharm J. 2002;268(7198):690.

18. Bayir PT, Demirkan B, Duyuler S, Guray U, Kisacik HL. Water intoxication resulting in ventricular arrythmias. Turkiye Acil Tip Dergisi. 2012;12(4):188-90.

19. Bennett M, Fitzpatrick G, Donnelly M. Rhabdomyolysis associated with polydipsia induced hyponatraemia. BMJ Case Rep. 2011;2011.

20. Boulanger-Gobeil C, St-Onge M, Laliberte M, Auger PL. Seizures and hyponatremia related to ethcathinone and methylone poisoning. J Med Toxicol. 2012;8(1):59-61.

21. Budisavljevic MN, Stewart L, Sahn SA, Ploth DW. Hyponatremia associated with 3,4-methylenedioxymethylamphetamine ("ecstasy") abuse. Am J Med Sci. 2003;326(2):89-93.

22. Carrascosa MF, Caviedes JR, Lucena MI, Cuadrado-Lavin A. Syndrome of inappropriate antidiuresis in doxylamine overdose. BMJ Case Rep. 2012;2012.

23. Chen HC, Chen CC, Chu P, Chao PC, Lin SH. Acute hyponatremic encephalopathy after preparation of colonoscopy. Journal of Medical Sciences. 2006;26(1):33-6.

24. Cho YS, Nam KM, Park JH, Byun SH, Ryu JS, Kim HJ. Acute hyponatremia with seizure and mental change after oral sodium picosulfate/magnesium citrate bowel preparation. Annals of Coloproctology. 2014;30(6):290-3.

25. Christenson LL, Scott D. Acute water intoxication following pelvic ultrasound examination. Postgrad Med. 1985;77(3):161-2.

26. Clark JM, Gennari FJ. Encephalopathy due to severe hyponatremia in an ultramarathon runner. West J Med. 1993;159(2):188-9.

27. Elsaesser TF, Pang PS, Malik S, Chiampas GT. Large-volume hypertonic saline therapy in endurance athlete with exercise-associated hyponatremic encephalopathy. J Emerg Med. 2013;44(6):1132-5.

28. Ferrer J, Halperin I, Conget JI, Cabrer J, Esmatjes E, Vilardell E. Acute water intoxication after intranasal desmopressin in a patient with primary polydispsia. J Endocrinol Invest. 1990;13(8):663-6.

29. Fisher A, Davis M, Croft-Baker J, Purcell P, McLean A. Citalopram-induced severe hyponatraemia with coma and seizure. Case report with literature and spontaneous reports review. Adverse Drug React Toxicol Rev. 2002;21(4):179-87.

30. Ghatol A, Kazory A. Ecstasy-associated acute severe hyponatremia and cerebral edema: a role for osmotic diuresis? J Emerg Med. 2012;42(6):e137-40.

31. Goodner DM, Arnas GM, Andros GJ, Waterhouse RB. Psychogenic polydipsia causing acute water intoxication in pregnancy at term. A case report. Obstet Gynecol. 1971;37(6):873-6.

32. Hiramatsu R, Takeshita A, Taguchi M, Takeuchi Y. Symptomatic hyponatremia after voluntary excessive water ingestion in a patient without psychiatric problems. Endocr J. 2007;54(4):643-5.

33. Hoffman MD, Stuempfle KJ, Sullivan K, Weiss RH. Exercise-associated hyponatremia with exertional rhabdomyolysis: importance of proper treatment. Clin Nephrol. 2015;83(4):235-42.

34. Hojer J. [Prognosis and treatment of symptomatic hyponatremia--a study of 28 intensive care cases]. Lakartidningen. 1992;89(37):2933-4, 9-40.

35. Iwazu Y, Honma S, Fujisawa G, Uki K, Yanaka I, Sato Y, et al. Hyponatremic seizure associated with acute respiratory infection. Clin Exp Nephrol. 2007;11(3):230-4.

36. Jackson KA. Acute symptomatic hyponatremia: Not just an ultraendurance phenomenon. Journal of Sports Chiropractic and Rehabilitation. 1996;10(2):67-70.

37. Karim MR, Jawairia M, Rahman S, Balsam L, Rubinstein S. Cocaine-associated acute severe hyponatremia. Clin Nephrol. 2011;75(SUPPL. 1):S11-S5.

38. Klonoff DC, Jurow AH. Acute water intoxication as a complication of urine drug testing in the workplace. JAMA. 1991;265(1):84-5.

39. Kuz GM, Manssourian A. Carbamazepine-induced hyponatremia: Assessment of risk factors. Ann Pharmacother. 2005;39(11):1943-6.

40. Leban V, Kozelj G, Brvar M. The syndrome of inappropriate antidiuretic hormone secretion after giant leaf frog (Phyllomedusa bicolor) venom exposure. Toxicon. 2016;120:107-9.

41. Ledochowski M, Kahler M, Dienstl F, Fleischhacker W, Barnes C. Water intoxication in the course of an acute schizophrenic episode. Intensive Care Med. 1986;12(1):47-8.

42. Lee LC, Noronha M. When plenty is too much: Water intoxication in a patient with a simple urinary tract infection. BMJ Case Rep. 2016;2016 (no pagination)(bcr-2016-216882).

43. Losonczy LI, Lovallo E, Schnorr CD, Mantuani D. Drinking to near death - Acute water intoxication leading to neurogenic stunned myocardium. Am J Emerg Med. 2016;34(1):119.e3-.e4.

44. Maclean D, Champion M, Trash DB. Pulmonary oedema during treatment of acute water intoxication. Postgrad Med J. 1976;52(610):532-5.

45. Madero M, Monares E, Dominguez AM, Ayus JC. Acute symptomatic hyponatremia in a flight attendant. Clin Nephrol. 2015;84(2):108-10.

46. Mukherjee S, Antonarakis ES, Asaduzzaman S, Peters JR. Acute psychological stress-induced water intoxication. Int J Psychiatry Clin Pract. 2005;9(2):142-4.

47. Nielsen JD. Water intoxication. [Swedish]. Lakartidningen. 1981;78(37):3162.

48. Odeh M, Oliven A. Coma and seizures due to severe hyponatremia and water intoxication in an adult with intranasal desmopressin therapy for nocturnal enuresis. J Clin Pharmacol. 2001;41(5):582-4.

49. Olivero JJ, Dichoso C. Severe hyponatremia in a home-dialysis patient. JAMA. 1978;239(2):108-9.

50. Putterman C, Levy L, Rubinger D. Transient exercise-induced water intoxication and rhabdomyolysis. Am J Kidney Dis. 1993;21(2):206-9.

51. Quinn CJ, Iyegha UP, Beilman GJ, Cerra FB. Acute correction of hyponatremia secondary to psychogenic polydipsia. American Journal of Case Reports. 2012;13:69-71.

52. Reynolds CJ, Cleaver BJ, Finlay SE. Exercise associated hyponatraemia leading to tonic-clonic seizure. BMJ Case Rep. 2012;2012.

53. Robert KM, Funk D, Margarites C. Acute postpartum altered mental status. Air Med J. 2008;27(3):136-8.

54. Rothwell SP, Rosengren DJ. Severe exercise-associated hyponatremia on the Kokoda Trail, Papua New Guinea. Wilderness Environ Med. 2008;19(1):42-4.

55. Scoccia B, Scommegna A. Carbamazepine-induced hyponatremia after transabdominal follicular ultrasound examination. Fertil Steril. 1988;50(6):984-5.

56. Severac M, Orban JC, Leplatois T, Ichai C. A near-fatal case of exercise-associated hyponatremia. Am J Emerg Med. 2014;32(7):813.e1-.e2.

57. Shibata K, Tsugawa S, Ohtsuji M, Kondo T, Ohshima T. Severe hyponatremia caused by cement ingestion during carbamazepine therapy. Am J Emerg Med. 1995;13(2):245-7.

58. Sonne DP, Overgaard-Steensen C. [Hyponatraemia as the cause of severe cerebral symptoms]. Ugeskr Laeger. 2013;175(39):2252-3.

59. Sonnenschein CH, Nielsen AK, Engquist A. [Cerebral symptoms in hyponatremia. Treatment and course]. Ugeskr Laeger. 1991;153(29):2065-9.

60. Sue YM, Lee YL, Huang JJ. Acute hyponatremia, seizure, and rhabdomyolysis after ecstasy use. J Toxicol Clin Toxicol. 2002;40(7):931-2.

61. Surgenor S, Uphold RE. Acute hyponatremia in ultra-endurance athletes. Am J Emerg Med. 1994;12(4):441-4.

62. Tilley MA, Cotant CL. Acute water intoxication during military urine drug screening. Mil Med. 2011;176(4):451-3.

63. Watson ID, Serlin M, Moncur P, Tames F. Acute hyponatraemia. Postgrad Med J. 1997;73(861):443-4.

64. Windpessl M, Schwarz C, Wallner M. "Bowel prep hyponatremia" - a state of acute water intoxication facilitated by low dietary solute intake: case report and literature review. BMC Nephrol. 2017;18(1):1-5.

65. Zelingher J, Putterman C, Ilan Y, Dann EJ, Zveibil F, Shvil Y, et al. Case series: Hyponatremia associated with moderate exercise. Am J Med Sci. 1996;311(2):86-91.

66. al-Zaki T, Jolly BT. Severe hyponatremia after "purification". Ann Emerg Med. 1997;29(1):194-5.

67. Glace B, Murphy C. Severe hyponatremia develops in a runner following a half-marathon. JAAPA : official journal of the American Academy of Physician Assistants. 2008;21(6):27-9.

68. Ofran Y, Lavi D, Opher D, Weiss TA, Elinav E. Fatal voluntary salt intake resulting in the highest ever documented sodium plasma level in adults (255 mmol L<sup>-1</sup>): A disorder linked to female gender and psychiatric disorders. J Intern Med. 2004;256(6):525-8.

69. Skjonnemand M. [A case of severe exercise-associated hyponatremia]. Ugeskr Laeger. 2013;175(19):1349-50.

70. Garigan TP, Ristedt DE. Death from hyponatremia as a result of acute water intoxication in an Army basic trainee. Mil Med. 1999;164(3):234-8.

71. Gutmann FD, Gardner JW. Fatal water intoxication of an Army trainee during urine drug testing. Mil Med. 2002;167(5):435-7.

72. Sjoblom E, Hojer J, Ludwigs U, Pirskanen R. Fatal hyponatraemic brain oedema due to common gastroenteritis with accidental water intoxication. Intensive Care Med. 1997;23(3):348-50.
